# A 2-step penalized regression method for family-based next-generation sequencing association studies

**DOI:** 10.1186/1753-6561-8-S1-S25

**Published:** 2014-06-17

**Authors:** Xiuhua Ding, Shaoyong Su, Kannabiran Nandakumar, Xiaoling Wang, David W Fardo

**Affiliations:** 1 Department of Biostatistics, University of Kentucky College of Public Health, 111 Washington Ave, Lexington, KY 40536-0003, USA; 2 Institute of Public and Preventive Health, Department of Pediatrics, Georgia Health Sciences University, School of Medicine, Georgia Prevention Center, HS-1640, Augusta, GA 30912, USA

## Abstract

Large-scale genetic studies are often composed of related participants, and utilizing familial relationships can be cumbersome and computationally challenging. We present an approach to efficiently handle sequencing data from complex pedigrees that incorporates information from rare variants as well as common variants. Our method employs a 2-step procedure that sequentially regresses out correlation from familial relatedness and then uses the resulting phenotypic residuals in a penalized regression framework to test for associations with variants within genetic units. The operating characteristics of this approach are detailed using simulation data based on a large, multigenerational cohort.

## Background

As biological techniques for assaying genetic variation have advanced, so have methods for candidate gene, genome-wide, exome, and whole genome association studies. In addition to methodological advancements purely statistical in nature, each progression has resulted in new analytical complexities, including those relating to problems of assuring reliable data quality and handling massive multiple testing and computational challenges. Increasingly, the ability to analyze the complex data resulting from genetic studies relies on having specialized software and vast computational resources. Methods are needed that are able to appropriately respect data complexity and are also accessible to investigators who wish to prevent an expensive computing investment.

Family-based association methodologies in the specific context of next-generation sequencing have been proposed [1]. Like any approach incorporating related individuals, these methods must somehow either utilize or, at the least, take into account, the dependence structure that necessarily exists within pedigrees. This within-family information can be drawn upon for the sake of inference as in transmission-based test statistics or, alternatively, can be adjusted for to remove any dependency that could violate independence assumptions of the downstream statistical test. When a goal is computational simplicity, in the context of large, complex pedigrees it could be advantageous to perform a within-family adjustment. The next-generation sequencing data set generated from the Genetic Analysis Workshop 18 (GAW18) contains more than 8 million variants from 20 complex pedigrees, and is thus ideal to examine these types of approaches.

The transition from designing genome-wide association studies (GWAS) that rest upon the common disease and common variant hypothesis to exome and whole genome studies that are better equipped to ascertain effects from rare variations has resulted in much interest for methods to group or aggregate variants in order to test for the multiple rare variant hypothesis that rarer variants of larger effect underlie common disease variation. In what follows, we present a method that handles complex pedigrees in a computationally accessible manner while also incorporating information over a genetic functional unit.

## Methods

### Data set

Whole genome sequencing for a subset of the San Antonio Family Studies (SAFS) participants was conducted through the Type 2 Diabetes Genetic Exploration by Next-generation sequencing in Ethnic Samples (T2D-GENES) Consortium. We examined the 1,215,399 variants on chromosome 3 that were genotyped on the 955 fully phenotyped subjects.

### Statistical analysis

The simple and fast way to test the effect of a genetic marker on a trait is to use a contrast of the frequencies or means among genotype groups in a linear model. However, this method does not explicitly take into account relationships among family members when available, which can lead to both false-positive and false-negative associations. The use of a linear mixed model is a potential solution to this problem when family-based data is available. These models adjust for familial relationships by modeling the polygenic component between individuals as a random effect but are computationally intensive. Recently, Aulchenko et al [2] developed a rapid and robust 2-step method based on a mixed-model framework for family-based association studies. The first step of this method is to perform a single polygenic analysis using the complete pedigree but ignoring marker data. Subsequently, the residuals from this analysis, which are now adjusted for polygenic covariance and fixed covariate effects, are used as an updated quantitative trait for association testing using classical methods for unrelated individuals. To our knowledge, however, this approach has not been adapted to rare variant testing. We adopted this 2-step polygenic regression adjustment and residual testing approach to test for both common and rare variants by inclusion of a penalized regression step. We then applied the method to the SAFS genome sequencing data.

*Step 1: adjustment for family structure*. First, we estimated the kinship matrix, Φ, in the SAFS pedigrees using a linkage disequilibrium-pruned subset of all common single-nucleotide polymorphisms (SNPs) genotyped in 955 participants [1,2]. For the sake of comparison, we also calculated the pairwise kinship coefficients based on the provided pedigree structure [3]. These 2 approaches for parameterizing relatedness were highly concordant as expected (correlation = 0.962; average difference 0.003 ± 0.006). Because genomic kinship coefficients do not depend on the completeness and quality of pedigrees and can provide more accurate information on ancestral relatedness than the given pedigree structure [1,4], we used genomic-estimated kinship to adjust for family structure in the following mixed model:

(1) $$y_{i}=\mu +\beta_{1}age_{i}+\beta_{2}gender_{i}+G_{i}+e_{i}$$

where *y_i _*is the phenotype of the *i*th individual, *μ *is a grand mean, $\beta_{1}$ and $\beta_{2}$ are the fixed effects of age and gender, and *G_i _*and *e_i _*are random additive polygenic and residual effects, respectively, for individual i. The vectors ***G ***and *e*, consisting of all polygenic and residual effects, follow zero-mean multivariate normal distributions with variance-covariance matrices $2\text{Φ}\sigma_{G}^{2}$ and $I_{n}\sigma_{e}^{2}$, respectively. Here $\sigma_{G}^{2}$ is the additive genetic variance explained by the kinship-based polygenic component, $I_{n}$ is an (*n × n*) identity matrix, *n *is the number of subjects, and $\sigma_{e}^{2}$ is the residual variance. The following vector of polygenic residuals, $y^{*}$, now has no dependence induced by familial relationships and was estimated as y*=σ^e2∑^-1(ê*), where ${ê}^{*}$ is the vector of trait values adjusted for covariates, that is, y-(μ^+β^1age+β^2gender), and ∑^ is the restricted maximum likelihood estimate of $2\text{Φ}\sigma_{G}^{2}+I\sigma_{e}^{2}$. These residuals were then used in the second step as quantitative traits from unrelated individuals. The R package GenABEL was used for this analysis [4].

*Step 2: penalized regression including a gene-based group penalty*. Instead of using the linear model in the original approach proposed by Aulchenko et al, we applied a lasso-type group penalized regression to better incorporate rare variants. This type of penalized regression has been applied to many forms of genetic analysis, including microarray data [5] and GWAS data [6]. Friedman et al [7] introduced the mixture of group and lasso penalties, and this approach has been explored using, for example, breast cancer GWAS data for testing common and rare variants [8]. In this study, variants were grouped by genes and assigned a weight, $s_{j}=2\sqrt{p_{j}\left(1-p_{j}\right)}$, where $p_{j}$ is the minor allele frequency for the *j*th SNP. The factor 2 makes the value of the weight range between 0 and 1. In our study, we adopted this method for the whole genome sequencing data with the residuals generated via step 1 from the family data. Briefly, we have an objective function to be minimized that comprises the underlying sums of squares from ordinary regression (first term) modified by a conventional lasso penalty and, finally, the group penalty, as below:

(2) $$f\left(\theta \right)=\frac{1}{2}||y^{*}-SNP\cdot \beta ||{\;}_{2}^{2}+\lambda_{L}\underset{j}{\sum }s_{j}\left|\beta_{j}\right|+\lambda_{E}\underset{G}{\sum }t_{G}{\left||\beta_{G}|\right|}_{2}$$

where ${||.||}_{2}$ denotes the L^2 ^norm, SNP is an n×m matrix of genotypes for the *m *SNPs, ***β ***is an m×1 vector of SNP effects, $\lambda_{L}$ and $\lambda_{E}$ are the lasso and group penalties, respectively, and $t_{G}$ is a weight function operating on the *G*th group, often used to adjust for gene size. Here, $\beta_{j}$ represents the (fixed) effect of the *j*th SNP, and $\beta_{G}$ corresponds to (fixed) effects for SNPs within the *G*th group, where for our purposes *G *is defined by gene. The use of the *L^2 ^*norm on the effects within a group encourages the incorporation of rare causal variants inside the same gene. To test the power of this approach on rare variants in particular, we further excluded common variants (minor allele frequency [MAF] ≥0.05) and repeated the analysis. These analyses were performed using the statistical software Mendel (Version 12.0) [9,10]. This version did not, to our knowledge, incorporate $t_{G}$ weighting, so its default of 1 was used throughout, giving equal weight to all genes.

## Results

We used GRCh37/hg19 build annotations to map chromosome 3 variants to genes; 521,355 of the 1,215,399 variants were located in a total of 1,165 genes. There are 188 causal variants, ie, variants with nonzero effect sizes for either diastolic blood pressure (DBP) or systolic blood pressure (SBP) at exam 1; these map to 31 unique genes. One hundred sixty-eight of these variants are causal for DBP and 134 for SBP. Genes with at least 1 causal variant are here referred to as *causal genes*. Table 1 presents SNP counts and other descriptors for each causal gene. Some variants used to simulate phenotypes lie nearby but outside of the genes assigned via the GAW simulation. As a result of these discrepancies, some causal genes include no mapped causal SNPs, an apparent contradiction. Interestingly, because we report on the proportion of simulation replicates that a SNP from a causal gene is in the final penalized regression model (Table 2), some causal genes with no causal SNPs are still detected. For example, *PAK2 *contains 4 SNPs used to model SBP and DBP, but, although none of these map within *PAK2 *using GRCh37/hg19, *PAK2 *can still be found in the fitted models.

**Table 1:** Causal gene characteristics.

	All variants					Variants with MAF <0.05				
	
	Variant counts		Causal	Total heritability		Variant counts		Causal	Total heritability	
	
Gene	Total	Causal	avg. MAF	DBP	SBP	Total	Causal	avg. MAF	DBP	SBP
*ABTB1*	48	2	0.08870	0.00070	0.00132	41	0	-	-	-
*ARF4*	161	0	-	-	-	108	0	-	-	-
*ARHGEF3*	2134	10	0.08890	0.00026	0.00007	1266	8	0.00986	0.00023	0.00006
*B4GALT4*	217	1	0.05110	0.00004	0.00002	127	0	-	-	-
*BTD*	291	8	0.00889	0.00041	0.00011	220	8	0.00889	0.00041	0.00011
*CXCR6*	0	0	-	-	-	0	0	-	-	-
*DNASE1L3*	115	7	0.14061	0.00023	0.00030	71	3	0.01900	0.00016	0.00025
*FBLN2*	687	4	0.02193	0.00021	0.00008	456	3	0.01073	0.00016	0.00006
*FLNB*	956	6	0.08682	0.00087	0.00280	636	5	0.00524	0.00002	0.00007
*GPR160*	244	2	0.21515	0.00004	0	165	1	0.00660	0.00001	0
*LOC152217*	7	1	0.07880	0.00002	0.00001	4	0	-	-	-
*MAP4*	894	15	0.06428	0.06483	0.07792	745	12	0.01048	0.05277	0.06336
*MLH1*	310	9	0.03816	0.00024	0	254	8	0.00409	0.00017	0
*MUC13*	203	6	0.05675	0.00022	0	128	4	0.00790	0.00014	0
*NMNAT3*	559	9	0.07731	0.00036	0.00031	386	6	0.00903	0.00019	0.00017
*PAK2*	819	0	-	-	-	524	0	-	-	-
*PDCD6IP*	466	5	0.19472	0.00061	0.00028	319	2	0.02140	0.00004	0.00002
*PPP2R3A*	1081	12	0.01400	0.00046	0.00010	881	11	0.00461	0.00046	0.00010
*PROK2*	66	3	0.25297	0.00045	0	46	0	-	-	-
*PTPLB*	493	3	0.02483	0.00007	0.00006	334	2	0.00410	0.00005	0.00004
*RAD18*	694	2	0.19195	0.00004	0	448	1	0.00820	0.00004	0
*RYBP*	347	4	0.18135	0.00046	0	301	2	0.01660	0.00002	0
*SCAP*	207	2	0.00835	0	0.00004	176	2	0.00835	0	0.00004
*SEMA3F*	134	2	0.01145	0.00004	0.00001	83	2	0.01145	0.00004	0.00001
*SENP5*	409	5	0.00788	0	0.00007	279	5	0.00788	0	0.00007
*SERP1*	18	1	0.34670	0.00001	0	10	0	-	-	-
*SUMF1*	747	3	0.06460	0.00010	0.00008	527	2	0.00410	0	0
*TFDP2*	1221	5	0.00824	0	0.00005	855	5	0.00824	0	0.00005
*TUSC2*	11	0	-	-	-	10	0	-	-	-
*VPS8*	1042	6	0.00748	0.00025	0	847	6	0.00748	0.00025	0
*ZBTB38*	590	9	0.15292	0.00059	0.00090	415	4	0.00408	0.00003	0.00003

Characteristics of the 31 causal genes (genes containing at least 1 causal variant as provided by the GAW18 simulation details). Total and causal variant counts are provided, as well as the average minor allele frequency for causal variants and the total gene-wide heritabilities for DBP and SBP. These metrics are provided using all variants in chromosome 3 (All variants) and after removing common variants (Variants with MAF ≥0.05). Discrepancies in mapping variants to genes between GRCh37/hg19 build annotations and the GAW18 simulations, result in some causal genes containing no mapped causal variants. Corresponding values for these genes are denoted by a dash but are retained for consistency.

**Table 2:** Detection probability for causal genes.

	DBP at exam 1				SBP at exam 1			
		
Gene	All variants		<5% MAF variants		All variants		<5% MAF variants	
				
	0.5	0.9	0.5	0.9	0.5	0.9	0.5	0.9
*ABTB1*	0.005	0.005	0	0	0	0.005	0	0
*ARF4*	0.005	0.005	0.025	0.025	0.020	0.020	0.035	0.035
*ARHGEF3*	0.025	0.125	0.110	0.185	0.030	0.130	0.100	0.155
*B4GALT4*	0	0	0.010	0.030	0	0	0	0
*BTD*	0.005	0.030	0.020	0.025	0	0.025	0.010	0.015
*CXCR6*	0	0	0	0	0	0	0	0
*DNASE1L3*	0.005	0.010	0.020	0.035	0.005	0.010	0.025	0.055
*FBLN2*	0.005	0.080	0.075	0.120	0.010	0.085	0.060	0.125
*FLNB*	0.035	0.110	0.095	0.130	0.120	0.360	0.100	0.140
*GPR160*	0	0.015	0	0.005	0	0.005	0	0
*LOC152217*	0	0	0	0	0	0.000	0	0
*MAP4*	0.995	1.000	1.000	1.000	0.990	0.995	1.000	1.000
*MLH1*	0.010	0.015	0.020	0.030	0	0	0	0.005
*MUC13*	0.005	0.015	0.065	0.120	0.005	0.020	0.045	0.100
*NMNAT3*	0.015	0.040	0.010	0.040	0.015	0.045	0.015	0.045
*PAK2*	0.005	0.045	0.030	0.055	0.010	0.035	0.030	0.060
*PDCD6IP*	0.010	0.015	0.015	0.020	0.010	0.020	0.015	0.015
*PPP2R3A*	0.005	0.005	0.035	0.045	0.010	0.015	0.025	0.025
*PROK2*	0.005	0.005	0.010	0.015	0	0	0.005	0.005
*PTPLB*	0.005	0.015	0.050	0.080	0.005	0.010	0.030	0.045
*RAD18*	0	0.035	0	0.005	0	0.020	0.005	0.005
*RYBP*	0.015	0.025	0.025	0.040	0.005	0.005	0.020	0.035
*SCAP*	0.180	0.185	0.260	0.260	0.150	0.155	0.210	0.210
*SEMA3F*	0.005	0.005	0.015	0.020	0	0	0	0
*SENP5*	0	0.010	0.030	0.075	0.005	0.015	0.010	0.020
*SERP1*	0	0	0	0.000	0	0.005	0.000	0
*SUMF1*	0	0.035	0.065	0.120	0	0.050	0.115	0.190
*TFDP2*	0.005	0.010	0.040	0.065	0	0	0.030	0.040
*TUSC2*	0	0	0	0	0	0	0	0
*VPS8*	0.005	0.030	0.050	0.095	0.015	0.020	0.090	0.130
*ZBTB38*	0.015	0.090	0.085	0.140	0.005	0.070	0.080	0.200

The proportion of the 200 simulation replicates that each causal gene (contains at least 1 variant with a DBP or SBP effect) is present in the selected in the final penalized regression model.

Each examined model specifies 134 predictors to be selected with nonzero regression coefficients using either a 50% or 90% weighting between the pure lasso and group penalties (ie, $\frac{\lambda_{L}}{\left(\lambda_{L}+\lambda_{E}\right)}=0.5\;\text{or}\;0.9$) and with either all variants or only those with MAF less than 5%.

**Figure 1:**
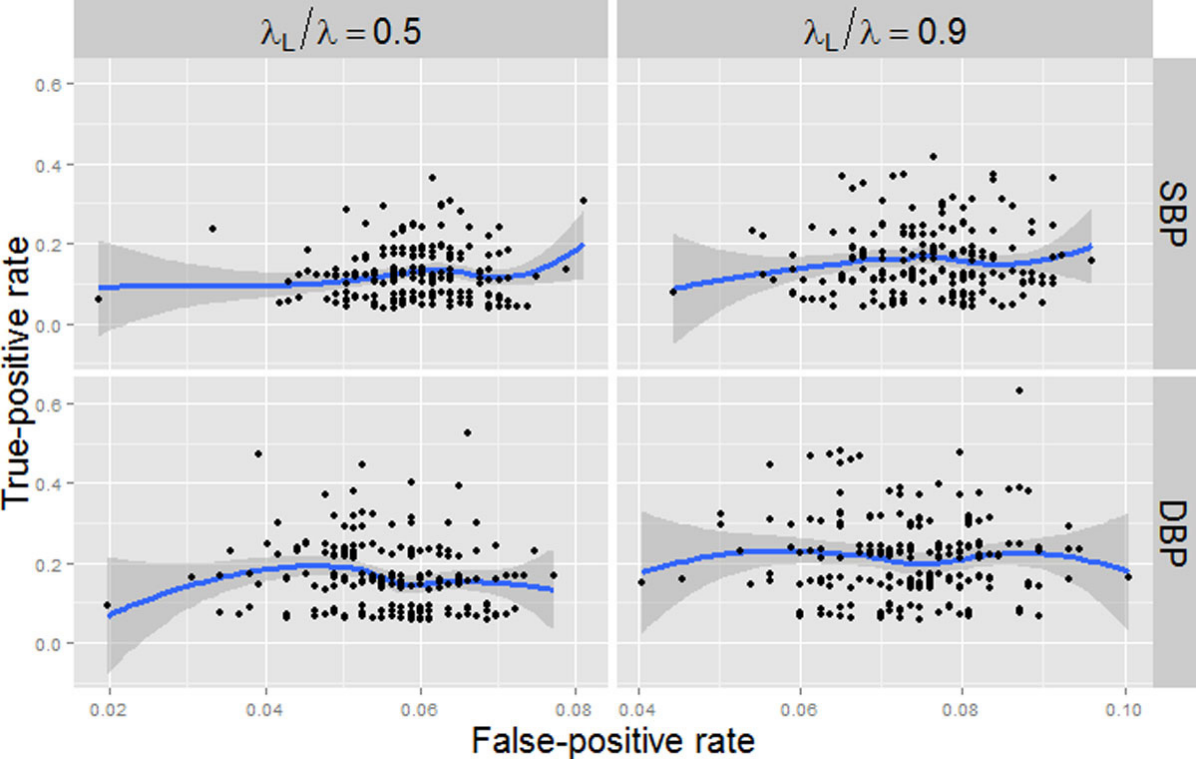
**Gene-based ROC curve**. Gene-based false-positive and true-positive rates plotted when employing a 50% and 90% lasso proportion on each trait (SBP and DBP) at exam 1 over the 200 simulation replicates. These plots are for models including all variants. Rates are defined as the number of distinct noncausal genes (genes containing no causal variants) in the final model divided by the total number of noncausal genes (False-positive rate; x-axis) and the number of distinct causal genes (genes with at least 1 causal variant) in the final model divided by the number of true causal genes (True-positive rate; y-axis). There are 27 (22) causal genes for DBP (SBP) and 1138 (1143) noncausal genes. Random "jitter" has been added to the y-axis because many replicates resulted in the same number of causal genes and a loess line with confidence bands illustrates a general trend. Results when using the 5% MAF restriction are similar, but with higher rates as a result of the decreased number of genes.

Table 2 provides the gene-based probabilities of discovering at least 1 SNP within each trait-specific (DBP or SBP) causal gene using either a 90% or a 50% lasso weight $\left(\frac{\lambda_{L}}{\left(\lambda_{L}+\lambda_{E}\right)}=\frac{\lambda_{L}}{\lambda }=0.5\right)$, as suggested by Zhou et al [8], and using all variants or only rare ones (MAF <0.05). For each model, we chose the regression penalty parameter to estimate the nonzero effects of approximately 134 variant predictors. This number was chosen for convenience from the number of causal SNPs for SBP. We examined other choices for this parameter (data not shown) and, even though not performing thorough cross-validation (a step that is common for penalized regression approaches but is not yet incorporated into Mendel), this appeared to provide a reasonable balance between capturing true and false positives in the final model. We examined how varying the relative contributions of the pure and group lasso penalties affects true-positive and false-positive rates. Figure 1 displays these rates for each of the 200 simulation replicates when testing all variants. Results when restricting to rare variants exhibit similar patterns, but have uniformly higher rates because of the exclusion of causal and noncausal genes with only common variants. An increase in the proportion of penalty for the pure lasso results in more distinct genes entering the final model, so both true-positive and false-positive rates are higher.

*MAP4 *is consistently discovered using our approach, which is not surprising because the variants within *MAP4 *confer more than 5% heritability for both DBP and SBP. No other gene encompasses that amount of heritability for either trait. In fact, the next highest heritability is that of *FLNB *for SBP (0.28%), and the other genes are correspondingly much less reliably detected. A few genes (eg, *ARHGEF3, FLNB*, and *SCAP*) are discovered with at least 10% probability in some models, although it is difficult to infer with confidence any pattern of characteristics for detection when these are so low. It appears that detection probability increases with more weight placed toward the pure lasso penalty, which is consistent with Figure 1. Restricting analysis to rare variants improves causal gene detection at least partially, as a result of the reduction in the number of genes considered, while retaining the same number of variant predictors in the model; for some genes (eg, *FLNB, MUC13, SCAP*, and *ZBTB38*) the improvement is considerable. Some interesting patterns in results from Table 2 can be attributed to gene characteristics in Table 1. For example, *FLNB *shows a markedly higher detection probability when testing SBP with all variants using a 90% lasso proportion; this discrepancy is because a common causal variant unique to SBP is removed from consideration when examining only common variants and is more reliably detected with pure lasso.

Per-gene false-positive rates are reasonably maintained (all below 0.03%), partially because of the upper bound constraint that the penalty tuning parameter imposes. That is, because only approximately 134 variants can enter the final regression model, there is a limit to the number of times each noncausal gene can be falsely discovered. False discovery rates (ie, the proportion of genes in the final models that are noncausal) are large, often above 90%, which is a known problem with penalized regression strategies.

## Discussion

Methods for handling complex pedigrees are varied and are often computationally intensive. The advent of next-generation sequencing exacerbates these complexities. We have proposed a novel method for approaching this problem that is easily implemented in freely available software packages and eases computational burden by regressing out correlations caused by familial relatedness in an initial step, preventing the estimation of often complex mixed models for each variant under consideration.

We chose to use a kinship matrix estimated based on genomic data to adjust for family structure because this confers several advantages over pedigree kinship [1,4,6]. First, it does not depend on the completeness and quality of the pedigree. For example, if one child is adopted but this information is not provided, the genomic kinship can give the correct estimation, while the pedigree kinship will classify the child as the first-degree relative of the parents and the sibs, which can induce bias. Second, genomic kinship may give a better estimate of a true covariance between individual genomes, while pedigree kinship provides only the expectation of the proportion of genome shared identical by descent. Third, genomic kinship can be incorporated in the presence of potential population stratification. Therefore, the use of genomic kinship is expected to lead to better estimates of polygenic model, and thus better power to detect association.

Methods that group variants within genes and treat the gene as a functional unit, as in the group lasso portion of our approach, can efficiently borrow information across the gene without necessarily testing the disease-influencing variants. We find that, in the context of the GAW18 simulated data, our method can successfully and consistently discover disease genes with sufficient heritability, but is largely underpowered when heritability is below 0.03%. More exploration of the proposed approach through simulation is warranted to examine cross-validation strategies for choosing lasso parameters, the related effects of linkage disequilibrium structure, and private variants. Notably, private variants (ie, those unique to a pedigree) will not likely be detected using our proposed methodology. The incorporation of tracking genetic transmissions will be required to do so, and any method that treats familial relatedness as a nuisance, as we do, in order to utilize test statistics for independent subjects will be severely underpowered for these variants.

## Competing interests

The authors declare that they have no competing interests.

## Authors' contributions

SS, XW, and DWF designed the overall study. XD, SS, KN, and DWF conducted the statistical analyses and created the tables and figure. SS and DWF drafted the manuscript, which was revised by XD, KN, and XW. All authors discussed the project throughout, read, and approved the final manuscript.
